# Auditory Nomenclature: Combining Name Recognition With Anatomical Description

**DOI:** 10.3389/fnana.2018.00099

**Published:** 2018-11-23

**Authors:** Bernd Fritzsch, Karen L. Elliott

**Affiliations:** Department of Biology, The University of Iowa, Iowa City, IA, United States

**Keywords:** ear, development, sensory epithelia, sensory neurons, auditory nuclei

## Abstract

The inner ear and its two subsystems, the vestibular and the auditory system, exemplify how the identification of distinct cellular or anatomical elements ahead of elucidating their function, leads to a medley of anatomically defined and recognition oriented names that confused generations of students. Past attempts to clarify this unyielding nomenclature had incomplete success, as they could not yet generate an explanatory nomenclature. Building on these past efforts, we propose a somewhat revised nomenclature that keeps most of the past nomenclature as proposed and follows a simple rule: Anatomical and explanatory terms are combined followed, in brackets, by the name of the discoverer (see Table 1). For example, the “organ of Corti” will turn into the spiral auditory organ (of Corti). This revised nomenclature build as much as possible on existing terms that have explanatory value while keeping the recognition of discoverers alive to allow a transition for those used to the eponyms. Once implements, the proposed terminology should help future generations in learning the structure-function correlates of the ear more easily. To facilitate future understanding, leading genetic identifiers for a given structure have been added wherever possible.

## Introduction

The ear was recognized as the organ for hearing since antiquity, but its function could only be understood mechanistically after Corti (1851) described some of the cells on the basilar membrane of what Kölliker soon referred to as the organ of Corti (Kölliker, 1852, 1867). Nearly overlapping in time, Reissner (1851) described the membrane separating the scala media from the scala vestibuli, now bearing his name (Reissner’s membrane) to identify three distinct channels in the cochlear canal instead of two as previously identified based on ever improving anatomical work. With the event of better preservation, decalcification and histological sections, many new features were discovered in the second half of 19th century. Naming those novel ear structures in the 1850-70 time frame continued a tradition of eponyms that dates back to Falloppio’s canal [now known as facial nerve canal (Politzer, 1907, 1981)] and followed the rational that names of first identifiers were associated with the structure they identified (Claudius, 1856; Boettcher, 1859; Hensen, 1863). Since discovery of new cell types outpaced for many years any reasonable understanding of their function, this approach was the most logical way forward to avoid over speculating on unclear function. In parallel to anatomical discoveries, functional ideas were proposed by Willis (1672). He believed that sound enters with movement of the stapes footplate through the oval window, is reflected and amplified in the semicircular canals before it is received by the “acoustic nerve” in the cochlea. Duverney (1730) noticed the different diameters of the cochlea duct and used his anatomical insights to invoke a resonance theory of hearing only much later elaborated on by Helmholtz (1859) and ultimately demonstrated as tonotopic organization of the cochlea by Békésy (1930). Neither name is in any way associated with their insights as eponyms, emphasizing the lopsided distribution of credit given by the somewhat random use of eponyms.

For example, it was only in 1789 that Scarpa (1800) surpassed the detailed description of Duverney (1730) and fully described the membranous labyrinth of the inner ear. And yet Scarpa’s name is only used as an eponym of the vestibular (or Scarpa’s ganglion; Table 1). The excellent illustrative work of von Sömmering (1806) which laid the foundation of much of the histology and comparative work of the 19th century, including the comparative work of Retzius (1881, 1884) never earned him any eponym. Even Retzius’ name was not associated with the amphibian papilla he described but is only associated with the Retzius’ bodies in the outer hair cells (Lim, 1986). After the foundation of the histology of the mammalian organ of Corti was established, details that were added later through more refined histological analysis did not earn eponyms such the newly described border cells for Held (1902, 1926). This contrasts sharply with the fact that Held’s earlier description of large contacts in brainstem auditory nuclei are now known under the eponym “endbulbs” and “calyx” of Held (1893).

**Table 1:** Terminology for the inner ear.

Latin terms	English terms	English terms	Molecular	Related terms
(TNA, 2017)	(US spelling,	(UK spelling;	signature	and Eponyms
	proposed terms)	TNA, 2017)		
**Cochlea**	**Cochlea**	Cochlea		
	Inner spiral sulcus			
	Outer spiral sulcus			
Modiolus cochleae	Modiolus	Modiolus		
Canalis spiralis modioli	Spiral canal of Rosenthal	Spiral canal of modiolus		Canal of **Rosenthal**
Canales longitudinales modioli	Longitudinal canals of modiolus	Longitudinal canals of modiolus		
Scala vestibuli	Vestibular scala	Scala vestibuli		
Helicotrema	Helicotrema	Helicotrema		Orifice of **Scarpa**
Scala tympani	Tympanic scala	Scala tympani		
**Ductus endolymphaticus**	**Endolymphatic duct**	Endolymphatic duct		
Saccus endolymphaticus	Endolymphatic sac	Endolymphatic sac		
Ductus reuniens	Ductus reuniens	Ductus reuniens		Duct of **Hensen**
**Ductus cochlearis**	**Middle duct**	**Cochlear duct**		Canal of **Reissner**
Membrana vestibularis	Vestibular membrane of Reissner	Vestibular membrane		Membrane of **Reissner**
Lamina basilaris	Basilar membrane	Basal lamina		Spiral membrane of **Duverney**
Membrana tectoria	Tectorial membrane	Tectorial membrane		
**Organum spirale**	**Spiral organ of Corti**	**Spiral organ**		Organ of **Corti**
**Cochleocytus**	**Hair cells**	**Hair cells**		Hair cells of **Corti**
Cochleocytus internus	Inner hair cell	Inner hair cell	*Fgf8*	
Cochleocytus externus	Outer hair cell	Outer hair cell	*Prestin*	
**Cellulae ductus cochlearis**		**Cells of cochlear duct**		
Epitheliocyti limitantes sulcus internus	Inner sulcus cells	Cuboidal inner sulcus cells		
Epitheliocytus limitans internus	Cnner border cell	Inner border cell	*GLAST, S100*	Inner border cell of **Held**
Epitheliocytus limitans externus	Outer border cell	Outer border cell		Outer border cell of **Hensen**
Epitheliocytus glandularis externus basalis	Outer glandular cell	Basal external glandular cell		Glandular cell of **Boettcher**
Epitheliocytus cuboideus sulcus externus	Outer sulcus cells	Cuboidal external sulcus cells	*BMP4*	Epithelial cell of **Claudius**
**Epitheliocyti sustenantes**	**Supporting cells**	**Supporting cells**		
Epitheliocytus internus pilae	Inner pillar cell	Internal pilar epithelial cell	*p75, Prox1*	Inner pillar cell of **Corti**
Epitheliocytus phalangeus internus	Inner phalangeal cell	Internal phalangeal epithelial cell	*GLAST, S100*	Inner phalangeal cells
Epitheliocytus externus pilae	Outer pillar cell	External pilar epithelial cell	*Prox1*	Outer pillar cell of **Corti**
Epitheliocytus phalangeus externus	Outer phalangeal cell	External phalangeal epithelial cell	*Prox1, S100, GLAST*	Epithelial cell of **Deiters**
Membrana reticularis	Reticular membrane	Reticular membrane		Reticular membrane of **Koelliker**
**Cuniculi**		**Tunnels**		
Cuniculus externus	Outer tunnel of Held	External tunnel		Tunnel of **Held**
Cuniculus internus	Pillar tunnel	Inner tunnel		Tunnel of **Corti**
Cuniculus intermedius	Outer phalangeal space	Intermediate tunnel		Space of **Nuel**
**Ganglion cochleare**	**Spiral ganglion**	**Cochlear ganglion**	*Neurod1, NeuN, TrkB, TrkC*	Ganglion cochleare of **Corti**
Perikaryon nonmyelinatum	Outer spiral ganglion neuron (oSGN)	Nonmyelinated perikaryon	*Peripherin, Th, Cgrp*	Type II neuron **of Spoendlin**
Perikaryon myelinatum	Inner spiral ganglion neuron (iSGNa,b,c)	Myelinated perikaryon	iSGNa = *Calb2* iSGNb = *Calb1* iSGNc = *Pou4f1*	Type I neuron **of Spoendlin**
Gliocytus ganglionicus ganglii cochlearis	Satellite cell of spiral ganglion	Satellite cell of cochlear ganglion	*Sox10, ErbB2*	
Neurofibra radialis ganglii cochlearis	Radial fiber of spiral ganglion	Radial fiber of cochlear ganglion		Mix of afferents and efferents
Fasciculus spiralis internus	Inner spiral bundle	Inner spiral bundle		
Fasciculus intraganglionicus	Intraganglionic spiral bundle	Intraganglionic spiral bundle	*AChE, Chna9, Chna 10*	Intraganglionic efferent bundle
Fasciculus spiralis externus	Outer spiral bundle	Outer spiral bundle		
**Ganglion vestibulare**	**Vestibular ganglion**	**Vestibular ganglion**		
Neuron bipolare ganglii vestibularis		Bipolar neuron of vestibular ganglion	*Neurod1, Pou4f1, TrkB*	Ganglion of **Scarpa (**with variable neuron size)
Gliocytus ganglionicus ganglii vestibularis		Satellite cell of vestibular ganglion	*Sox10, ErbB2*	
**Nervus vestibulocochlearis**	**Vestibulocochlear nerve**	**Vestibulocochlear nerve**		
**Nervus vestibularis**	**Vestibular nerve**	**Vestibular nerve**		
Ramus communicans cochlearis	Vestibulocochlear anastomosis	Cochlear communicating branch	*AChE, Chna9*	Vestibulocochlear anastomosis of **Oort**
Pars superior	Utriculoampullary nerve	Superior part		**Related term:** Nervus vestibularis superior.
Nervus utricularis	Utricular nerve	Utricular nerve		
Nervus ampullaris anterior	Anterior ampullary nerve	Anterior ampullary nerve		
Nervus ampullaris lateralis	Lateral ampullary nerve	Lateral ampullary nerve		
Pars inferior	Inferior part	Inferior part		
Nervus ampullaris posterior	Posterior ampullary nerve	Posterior ampullary nerve		**Related term:** Nervus vestibularis inferior.
Nervus saccularis	Saccular nerve	Saccular nerve		**Related term:** Nervus vestibularis posterior.
**Nervus cochlearis**	**Auditory nerve**	**Cochlear nerve**		**Related term:** Nervus auditus.

*This table was modified after (FCOA, 1998; FIPAT, 2017)*.

Many years of continued insight into the cellular and subcellular details of the organ of Corti, organization and function allow now to go beyond the purely descriptive and initially disputed original work. Today, the entrenched use of eponyms in otolaryngology confuses students and blocks understanding through enforced learning of eponyms that have no meaning beyond honoring the original descriptor and conserve an anatomical terminology that is in part unrelated to the function that was mostly unclear at the time the structures were first described. Eponyms were less fashionable from 1880 to today, novel features nevertheless received trivial names that do not convey the level of understanding detailed anatomy, physiology and molecular development of the ear now allows. Inconsistencies abound, such as the inner border cells [Grenzzelle (Held, 1902)] are not called Held’s cells whereas the outer border cells are now referred to as Hensen’s (1863) cells. Likewise, the outer phalangeal cells are now mostly referred to as Deiters (1860) cells whereas the inner phalangeal cells have no eponym. Complicating cochlear nomenclature even further, some trivial names are redundant and confusing such as type 1 and 2 hair cells in the vestibular system and Type I and II spiral ganglion neurons in the cochlea, evoking false associations in students new to the ear nomenclature. And some names were differently translated such as the German “Pfeilerzelle” is now referred to in US English as “pillar cells” but in United Kingdom English as “pilar cells,” with only the former presenting a translation according to the German meaning. Some of these issues have been partially rectified by taking traditional/scientific terms, multilingual discrepancies, role of Latin terms, usage of adjectives vs. genitive, usage of poorly defined words, usage of eponyms into account in previous nomenclature revisions (FCOA, 1998; FIPAT, 2017). The motivation for the present revision is to build on these past considerations reflected in the most recently proposed nomenclature (Table 1) while taking a more novel molecular and functional considerations into account.

Obviously, eponyms avoided associating mistaken functions to various parts of the ear (Politzer, 1907, 1981; Lustig et al., 1998; Mudry, 2001) and isolated the morphological description from functional speculations, certainly an important consideration at a time when vestibular and auditory function of the ear were mostly unknown and in many cases simply misinterpreted. Adding to this confusion in the more recent literature were mistranslations [the border cells of Held are now mostly referred to as “inner border cells” (Held, 1902) due to a mistake in one summary image] that identified what appears to be the same cell by different names. It was only later that hearing and vestibular function could be associated with different parts of the ear through the works of Mach (1865a,b), Breuer (1873), Barany (1906), Békésy (1930) and Helmholtz (1859). Both the function of the ear as a gravistatic and angular motion detection system and the function of the cochlea as a frequency and intensity monitoring system have been clarified as distinct functions of the mammalian ear (Hudspeth, 1989). The detailed understanding of the organ of Corti was advanced by modern techniques beyond the excellent description of Held (1902, 1926) using electron microscopy, summarized by Lim (1986) and Slepecky (1996) and quantitative ratios of different cell types of the organ of Corti (Jahan et al., 2015). We now know that hair cells function as polarized mechanotransducers (Hudspeth, 1989) with a distinctly different function of the inner and outer hair cells in amplification and reception of sound (Zheng et al., 2000). For example, sound stimulation of the organ of Corti was long been depicted as a simple up-down movement that directly caused shearing forces of the tectorial membrane on the inner hair cell stereocilia (Lewis et al., 1985). In contrast, more recent work suggest that the adult inner hair cell is not connected to the tectorial membrane (Lim, 1986) but acts as a hydrodynamic receptor monitoring endolymph flow in and out of the subtectorial space (Elliott et al., 2018).

More recent work on early development using gene expression and functional assessments of afferent, efferent, and hair cell proteins provides novel ways of identifying cells of the ear not only based on their topology and function but on their molecular signature (Liu et al., 2014). Unsurprisingly, such molecular data open again issues of identification of cell types and regrouping previous anatomical distinctions into smaller subgroups. For example, spiral ganglion neurons were initially described as homogenous (Corti, 1851) or as multiple types (De No, 1981), regrouped eventually into just two types based on diameters and innervation (Spoendlin, 1971), but subsequently again expanded to three types based on physiological properties (Merchan-Perez and Liberman, 1996; Rutherford and Moser, 2016). The latter suggestions are now supported by their molecular signatures (Petitpré et al., 2018; Shrestha et al., 2018; Sun et al., 2018). While all papers agree on the major expression they use inconsistent, albeit similar nomenclature: for example, what is Type Ia in two papers (Shrestha et al., 2018; Sun et al., 2018) is Type Ic in the third paper (Petitpré et al., 2018). The solution to this emerging nomenclature problem is to adopt a more meaningful nomenclature such as inner Spiral Ganglion Neurons, subtype a (iSGNa) as proposed in Table 1. It is to be expected that further single cell sequencing will likely lead to subdivisions of vestibular ganglion neurons as well given their cellular heterogeneity.

While some genes such as Sox2 are associated early in development with all neurosensory cells of the ear, they later become restricted to supporting cells following upregulation of high levels of Atoh1 in hair cells (Dabdoub et al., 2008). Interestingly enough, such gene expression over time depends on the level of expression of other transcription factors, as inner pillar cells show only limited expression of Atoh1 that does not affect Sox2 expression (Matei et al., 2005). Thus, while anatomical features and their physiological implications are largely settled, molecular signatures are still in flux due to technical advances that permit cell specific expression profile assessment to understand the complex cell type development and maintenance (Booth et al., 2018) as well as the gene expression profiles leading to specific structures such a stereocilia development (Ellwanger et al., 2018). Past research has stepwise improved the understanding of how sound moves the basilar membrane/organ of Corti/tectorial membrane complex to provide topology specific amplification (Ren et al., 2016; Dewey et al., 2018), including a detailed understanding of the function of the cochlear amplifier in the three rows of outer hair cells (Xia et al., 2018). Increasingly detailed insights into the function of the various sections of the organ of Corti have revealed major distinctions as an outer section playing a role in sound amplification and an inner section playing a role in sound conversion (Elliott et al., 2018). Molecular signatures that highlight nearly all outer section cells, including the inner pillar cells, such as Prox1, have been described (Fritzsch et al., 2010) that set the organ of Corti apart from vestibular sensory epithelia (Bermingham-McDonogh et al., 2006). Other transcription factors are uniquely found in a single cell type of the organ of Corti such as Fgf8 in inner hair cells that is found in many vestibular hair cells (Jahan et al., 2018) or the p75 neurotrophin receptor in inner pillar cells but also in sensory neurons (Von Bartheld et al., 1991). As more single cell transcriptome analyses are published, the current insights will likely be supplemented by both better characterization of unique expression profiles but will likely also end up indicating that some specificity is only a matter of thresholds of detection inherent to applied techniques.

## Goals of the Proposed Revision

**FIGURE 1:**
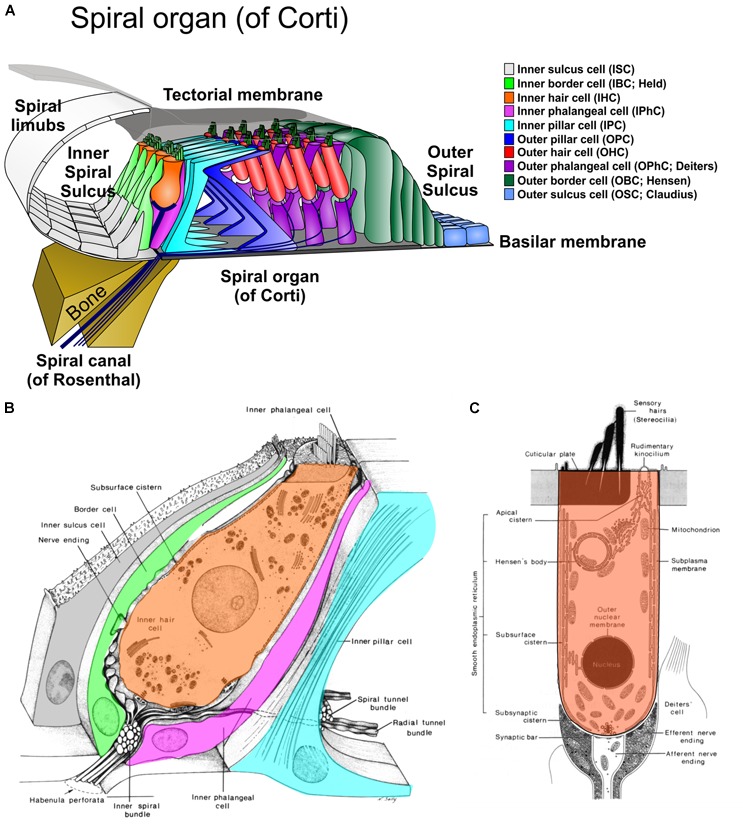
The cellular organization of the organ of Corti is shown in a radial section **(A)** and the details of the inner hair cell **(B)** and outer hair cell **(C)** as revealed by transmission electron microscopy. Radial sections suggest a simple numerical relationship of cells of the inner section (1IBC, 1IHC, 1IPhC, 1 IPC) and other section (1OPC, 3 OHC, 3 OPhC, 3+ OBC). Note that the inner pillar cell (IPC) sits on the bony lip of the spiral canal (of Rosenthal). Equivalent cells of the outer and inner section are in different shades of the same color. Modified after Elliott et al. (2018) and Lim (1986).

**FIGURE 2:**
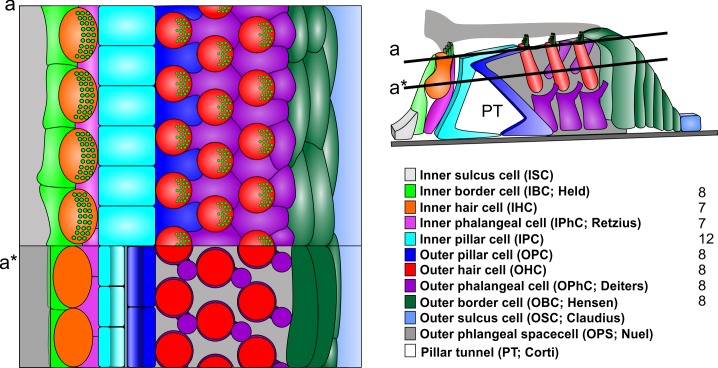
In contrast to radial sections, top views on the reticular lamina **(a)** or horizontal sections below the reticular lamina **(a^∗^)** indicate a different numerical ration between the cells of the inner section and outer section (right). Note that the largest number of cells are the iIPC with no clear numeric ratio to any of the adjacent cells. Note that the outer section has a simple 1:1 ratio between all elements of a given row also the details of most cells differ. For example, the reticular lamina is formed by the out rudder of the OPC between the first row of OHC (blue) but by the 1+2 second rows of OPhCs between the 2+3 row of OHC. The third row of OPhCs forms a continuous boundary along the reticular lamina flanking OBCs. IHC are in direct contact to IPCs only at the reticular lamina **(a)** whereas they are in contact with each other below the reticular lamina and are completely separated from IPC by the IPhC. Modified after Jahan et al. (2015).

With this caveat of some future refinement based on deeper molecular understanding in mind, we propose here a revision of the most recent nomenclature (FCOA, 1998; FIPAT, 2017) that primarily builds on topology, physiology and, wherever possible, unique molecular signature (Figures 1, 2 and Table 1), taken ultrastructural details and their functional significance revealed over the last 70 years into account (Engström et al., 1964; Kimura, 1975; Lim, 1986; Slepecky, 1996). We propose to divide the spiral auditory organ (of Corti) into an **inner and an outer section** with appropriate expansion of the existing nomenclature to name each element accordingly:

**The inner section** is the sound receiving section. We propose to expand the already partially consistent nomenclature (inner spiral sulcus, inner hair cell, inner pillar cell, inner phalangeal cells (FCOA, 1998; FIPAT, 2017)) that excludes some other relevant features. For example, it is now clear that the two major types of spiral ganglion neurons, type I and II, innervate the inner and outer hair cells, respectively. We therefore propose to use a new nomenclature of inner spiral ganglion neuron and outer spiral ganglion neuron instead of type I and type II. With the exception of transient expansion of some inner spiral neurons into the outer section during development (Druckenbrod and Goodrich, 2015; Goodrich, 2016) and under certain conditions of hair cell disorganization (Jahan et al., 2018), these neuronal processes of type 1 spiral ganglion neurons remain within the inner section and are named inner spiral ganglion neurons. Beyond possible transient developmental expansions to outer hair cells, the so-called lateral olivo-cochlear (LOC) system of inner ear efferents (Simmons et al., 2011) remains also restricted to the inner section and should thus be referred to as the inner (olivo-cochlear) efferents. Past use was also inconsistent with respect to (inner) border cells, dating back to the original description of this cell (Held, 1902) and extending into more detailed histology (Lim, 1986; Slepecky, 1996). We propose to use inner border cells to highlight these transitional cell type from the inner sulcus cells and propose to use the term outer border cells for the transitional cell type to outer sulcus cells, both with appropriate eponyms [inner border cells (of Held), outer border cells (of Hensen)].

**The outer section** is the sound amplifying section. The nomenclature of this section is less consistent overall (FCOA, 1998; FIPAT, 2017). The outer pillar cells (of Corti) and outer sulcus cells (of Claudius) are in the existing nomenclature as well as outer phalangeal cells (of Deiters). Neither the Hensen cells (here referred to as outer border cells of Hensen) nor the Boettcher cells (restricted to the basal turn) have been included into a consistent nomenclature. As with the inner section, both afferent and efferent innervation can be renamed to reflect their exclusive projection to outer hair cells in the adult organ (Rubel and Fritzsch, 2002; Goodrich, 2016). With this exclusive connection in normal adult mammals in mind, type II spiral ganglion neurons should be renamed as outer spiral ganglion neurons. Likewise, the clear exclusive connection of the medial olivo-cochlear neurons to outer hair cells (Simmons et al., 2011) necessitates to rename them as outer (olivo-cochlear) efferents. Note that this nomenclature proposal for afferent and efferent neurons reflects to terminals in spiral auditory organ (of Corti) and not the distribution of their cell bodies near the superior olivary complex as in the past.

Adopting this nomenclature would help to entrench the functional differences of the two sections in the context of their topology: the **inner section is the “hearing” section** that has all the inner hair cells with associated inner supporting cells, inner afferents and inner efferents needed for hearing. In contrast, the **outer section is the “amplifier” section** with the contractile outer hair cells innervated predominantly by the outer efferents with outer spiral afferents playing a role only in very loud sound hearing related to damage (Liu et al., 2015). Both sections are mirror symmetric with respect to cell type distribution.

The **inner section** cell types progresses from medial (modiolar) to lateral as follows: inner sulcus cells (ISC), inner border cells (IBC), inner hair cells (IHC), inner phalangeal cells (IPhC), inner pillar cells IPC (Figure 1).

The **outer section** cell types progresses (in reverse cellular order) from lateral to medial as follows: outer sulcus cells (OSC), outer border cells (OBC), outer hair cells (OHC), outer phalangeal cells (OPhC), and outer pillar cells (OPC; Figure 1). The pillar tunnel (of Corti) divides the numerical and organizationally distinct (Jahan et al., 2015) inner and outer section.

While the two sections have similar overall numbers of cell types (excluding the basal outer border cells [of Boettcher] in the apex, the total numbers of cellular units to each section vary dramatically. For example, the inner section receives the vast majority of afferents (∼95%) and efferents (∼60%) but has overall fewer units of each cell type in a radial section (one IBC as compared to 2–4 OBC, one IPhC as compared to three OPhC, one IHC compared to three OHC [except for reduced numbers in the base and increased numbers in the apex]. The only symmetry in terms of numbers of elements are IPC and OPC. However, this apparent symmetry even of these cells is a consequence of the radial section perspective (Figure 1). Viewed from the reticular lamina, the OHC and OphC/OPC form a nearly perfectly alternating cellular network (Figure 2). In contrast, near the basal lamina, all supporting cells in the outer section are in broad contact with each other without any outer hair cell in between. Interestingly enough, while IPC and OPC are in broad contact both basally and apically (Lim, 1986; Slepecky, 1996), the numbers of IPC and OPC cells are in a 3:2 ratio (Held, 1902). Whereas OHC are never in contact with each other, IHC are in very broad contact with each other being separated only at the reticular lamina by the IPhC and IBC [Lim, 1986; Held, 1902; Slepecky, 1996] and touching only at the reticular lamina the IPC (Figures 1, 2). Thus, while lateral inhibition with the delta–notch interaction may explain the formation of the outer section mosaic it fails to explain the inner section cell assembly. In fact, the real numerical relationship of each cell type for a given stretch of the spiral auditory organ (of Corti) for humans is: IBC = 8; IHC = 7; IPhC = 7; IP = 12; OP = 8; IHC = 8 × 3 rows; OPhC = 8 × 3 rows; OBC = 8 × 3 - 4 rows (Jahan et al., 2015).

While some of these odd numerical relationships have been known since Retzius (1884) and Held (1902) counted them, their implication for developmental biology in terms of regulating their differential numbers has been nearly universally ignored. Various studies have revealed that this ratio is extremely dependent on diffusible factors and cell–cell interactions (Groves and Fekete, 2012, 2017; Jahan et al., 2018). More recent emphasis on effects of gene replacement on these cellular numeric ratios and their distribution have re-emphasized these differences between the two sections that need to be understood for any forward looking strategy to restore a functional spiral auditory organ (of Corti) and thus hearing from a flat epithelium (Jahan et al., 2018). Restoring an outer section will certainly not restore hearing but an inner section associated with proper amplification might be beneficial to maintain most afferent innervation through neurotrophic support (Fritzsch et al., 2016) and might be useful for hearing with proper amplification to offset the loss of the outer section. Overall, our proposal takes much of the existing nomenclature (FCOA, 1998; FIPAT, 2017) into account but provides a more uniform description of cellular elements around the now understood functional sections of the spiral auditory organ (of Corti), the mammalian hearing organ.

## Author Contributions

BF conceived and wrote the initial draft. KE reviewed the draft and prepared the illustrations.

## Conflict of Interest Statement

The authors declare that the research was conducted in the absence of any commercial or financial relationships that could be construed as a potential conflict of interest.
